# Targeting the PCSK9–PR–IL-17 axis for psoriasis therapy

**DOI:** 10.3389/fphar.2026.1884619

**Published:** 2026-09-25

**Authors:** Yan Li, Yingxue He, Shengwei He, Zirun Yin, Kai Huang, Chenyang Jiao, Yugui Xia, Qiong Luo, Chao Luan, Wenjie Guo

**Affiliations:** 1 State Key Laboratory of Pharmaceutical Biotechnology, Department of Gastroenterology, Nanjing Drum Tower Hospital, School of Life Sciences, Nanjing University, Nanjing, China; 2 Institute of Artificial Intelligence Biomedicine, Nanjing University, Nanjing, China; 3 Jiangsu Key Laboratory of Molecular Biology for Skin Diseases and STIs, Institute of Dermatology, Chinese Academy of Medical Sciences and Peking Union Medical College, Nanjing, China

**Keywords:** IL-17 signaling, imiquimod model, keratinocyte hyperproliferation, progesterone receptor, proprotein convertase subtilisin/kexin type 9, psoriasis pathogenesis

## Abstract

**Introduction:**

Psoriasis is a chronic, immune-mediated inflammatory dermatosis characterized by keratinocyte hyperproliferation and systemic metabolic comorbidities. Although proprotein convertase subtilisin/kexin type 9 (PCSK9) is a well-established regulator of lipid metabolism, its role in the cutaneous inflammatory microenvironment remains poorly defined. This study investigated the contribution of PCSK9 to psoriatic inflammation and its potential underlying mechanisms.

**Methods:**

PCSK9 expression was assessed in human psoriatic lesions and an imiquimod (IMQ)-induced mouse model. The effects of pharmacological PCSK9 inhibition with SBC-110736 were evaluated using PASI-like scores and inflammatory cell infiltration. Transcriptomic analysis was performed to identify associated signaling pathways. The relationship between PCSK9 and progesterone receptor (PR) expression in keratinocytes and the effects of the PR agonist progesterone were also examined.

**Results:**

PCSK9 expression was significantly upregulated in human psoriatic lesions and in the IMQ-induced mouse model. SBC-110736 attenuated psoriasis-like disease severity, reducing PASI-like scores and infiltration of CD4^+^ and CD11b^+^ cells. Transcriptomic analysis indicated that PCSK9 inhibition primarily suppressed interleukin-17 (IL-17) signaling while restoring Pgr expression. PCSK9 and PR expression were inversely associated in keratinocytes, suggesting that PCSK9-mediated PR downregulation may exacerbate IL-17-driven inflammation. Progesterone treatment produced similar therapeutic effects to those observed with PCSK9 inhibition.

**Discussion:**

These findings suggest that a PCSK9–PR–IL-17 axis contributes to psoriasis pathogenesis, with PR potentially serving as an endogenous anti-inflammatory brake. Targeting this pathway may offer a strategy to modulate cutaneous inflammation, while its potential effects on related metabolic disturbances warrant further investigation.

## Introduction

Psoriasis is a chronic, immune-mediated systemic disease affecting approximately 2% of the global population (Langley et al., 2005). Clinically, it presents as well-demarcated erythematous plaques with silvery scales, reflecting pathological crosstalk between immune cells and keratinocytes (Lowes et al., 2014). Beyond cutaneous manifestations, psoriasis is increasingly recognized as a systemic disorder frequently comorbid with metabolic syndrome, dyslipidemia, and insulin resistance, and is associated with an elevated risk of cardiovascular disease (Grayson, 2012; Mrowietz et al., 2024). These associations suggest overlapping inflammatory pathways linking skin inflammation and metabolic dysregulation. The immunopathogenesis of psoriasis centers on the IL-23/IL-17 axis. Dendritic cells and macrophages produce IL-23, which promotes the differentiation and expansion of T helper 17 (Th17) cells and other innate lymphoid populations that secrete IL-17A. IL-17A acts on keratinocytes to drive hyperproliferation, induce antimicrobial peptides, and elicit proinflammatory chemokines, thereby amplifying immune cell recruitment and inflammation (Nestle et al., 2009). Despite the success of biologics targeting this pathway, many patients have an inadequate response, develop loss of response over time, or face limited access due to cost, underscoring the need for additional therapeutic targets and mechanisms (Armstrong and Read, 2020). Proprotein convertase subtilisin/kexin type 9 (PCSK9) is best known for regulating cholesterol homeostasis by binding the low-density lipoprotein receptor (LDLR) and promoting its lysosomal degradation, thereby increasing circulating low-density lipoprotein cholesterol (Seidah et al., 2014). PCSK9 inhibitors are established therapies for hypercholesterolemia and cardiovascular disease (Jiang et al., 2025). Beyond lipid regulation, accumulating evidence supports pleiotropic roles of PCSK9 in inflammation and immunity, including modulation of cytokine production, immune cell activation, and endothelial function (Bao et al., 2024). In psoriasis, clinical studies report increased serum and lesional PCSK9 levels that correlate with disease severity, and small studies suggest that systemic PCSK9 inhibition may improve skin disease independent of lipid lowering (Luan et al., 2019; Merleev et al., 2022; Garshick et al., 2021). However, whether PCSK9 acts locally in skin and the molecular mechanisms by which it exacerbates psoriatic inflammation remain unclear.

Given the skin’s capacity for local steroidogenesis and sex hormone signaling, we hypothesized that PCSK9 may interfere with endogenous hormonal checkpoints that normally restrain inflammation (Hannen et al., 2011). Progesterone receptor (PR) signaling is an important modulator of immune responses in multiple tissues, with anti-inflammatory effects reported in models of autoimmune and inflammatory disease (Tait et al., 2008; Cui et al., 2022). In skin, PR signaling has been implicated in barrier maintenance, wound healing, and immune modulation, but its role in psoriasis has not been systematically examined (Murakami et al., 2022; Im et al., 2000).

In this study, we investigated the local expression and function of PCSK9 in psoriatic skin and explored the underlying mechanism. Using patient samples, an imiquimod (IMQ)-induced mouse model, and keratinocyte cultures, we show that PCSK9 is upregulated in psoriatic lesions and promotes inflammation by suppressing PR expression, thereby enhancing IL-17-mediated signaling. We further show that pharmacological PCSK9 inhibition and PR activation ameliorate psoriasis-like pathology *in vivo* and suppress IL-17 responses *in vitro*. Collectively, our findings delineate a PCSK9–PR–IL-17 regulatory axis in skin that links a metabolic regulator to cutaneous inflammation and provide a mechanistic rationale for targeting this pathway in psoriasis.

## Materials and methods

### Antibodies

Anti-PCSK9 (sc-55206), anti-PR (sc-398898), and anti-PCNA (sc-53407) antibodies were purchased from Santa Cruz Biotechnology (United States). Anti-DYKDDDDK tag polyclonal antibody (20543-1-AP), phospho-NF-κB p65 (Ser536) recombinant monoclonal antibody (80379-2-RR), and Ki-67 polyclonal antibody (28074-1-AP) were obtained from Proteintech (United States). Anti-β-tubulin antibody (M20005) was purchased from Abmart (China). Alexa Fluor 488 anti-mouse CD4 antibody (100425) and Alexa Fluor 594 anti-mouse/human CD11b antibody (101254) were purchased from BioLegend (United States).

### Reagents

Imiquimod (Aldara) was purchased from 3M Health Care Limited (United Kingdom). SBC-110736 (HY-101832), Carbomer 940 (HY-W250721A), glycerol (HY-B1659), progesterone (HY-N0437), and a protease and phosphatase inhibitor cocktail (HY-K0013) were obtained from MedChemExpress (United States). Recombinant human IL-17A (C021) was purchased from Novoprotein (China). Hieff Trans Universal Transfection Reagent (40808ES02) and phenylmethylsulfonyl fluoride (PMSF; 20104ES03) were purchased from YEASEN (China). The immunohistochemistry (IHC) detection kit (DAB, PR30018) was purchased from Proteintech (China). The reverse transcription kit (R323-01) and SYBR Green PCR Master Mix (Q321-02) were purchased from Vazyme (China). Fetal bovine serum and Dulbecco’s Modified Eagle Medium (DMEM) were purchased from Biological Industries (Israel). RIPA lysis buffer (P0013K), DMSO (ST038), PEG400 (Y268728), Tween 80 (ST2789), vitamin C (Y268174), and penicillin-streptomycin solution (100 μg/mL streptomycin and 100 U/mL penicillin; C0222) were purchased from Beyotime (China). Triethanolamine (V900257) was purchased from Sigma-Aldrich (United States). RNAiso Plus (9109) was purchased from Takara (Japan). The BCA assay kit (23227) and Opti-MEM reduced-serum medium (11058021) were obtained from Thermo Fisher Scientific (United States).

### Cell culture

HaCaT cells (SCSP-5091, National Collection of Authenticated Cell Cultures, China) were cultured in DMEM supplemented with 10% fetal bovine serum and 1% penicillin-streptomycin. Cells were maintained at 37 °C in a 5% CO_2_ incubator (Thermo Fisher Scientific, United States).

### IMQ-induced psoriasis-like mouse model

Eight-week-old female or male C57BL/6J mice were purchased from GemPharmatech (Nanjing, China) and used to establish an IMQ-induced psoriasis-like dermatitis model. Mice received a daily topical application of 62.5 mg of 5% imiquimod cream on the shaved dorsal skin for four consecutive days, while control mice received the same amount of vehicle cream.

### Drug treatment

For pharmacological intervention, SBC-110736 was administered intraperitoneally once daily at 10 mg/kg, dissolved in a vehicle composed of 10% DMSO, 30% PEG 400, and 5% Tween 80 in phosphate-buffered saline (PBS), to achieve sufficient systemic exposure for PCSK9 inhibition. A gel containing 0.1% or 0.3% progesterone was applied topically to the dorsal skin once daily to facilitate local PR activation in psoriatic skin lesions. The gel was prepared with Carbomer 940 (0.8%), glycerol (20%), vitamin C (1%), and deionized water to 100 mL, and the pH was adjusted to 6–7 with triethanolamine. All treatments were initiated on the day after the first IMQ application and continued until euthanasia. Body weight and skin condition were recorded daily before drug administration.

Male mice were used for progesterone treatment to minimize the potential influence of estrous-cycle-dependent fluctuations in endogenous ovarian hormones on the assessment of exogenous progesterone responses. This design was intended to reduce hormone-related variability during short-term pharmacological intervention.

For cell stimulation, HaCaT cells were co-treated with recombinant human IL-17A (50 ng/mL) and progesterone (0, 1, or 3 μM) for 24 h.

### Histology, immunohistochemistry (IHC), and immunofluorescence (IF)

Harvested mouse dorsal skin and human skin tissues were rinsed with PBS, fixed in 4% formaldehyde overnight, and embedded in paraffin. Sections (4 µm) were cut for hematoxylin and eosin (H&E) staining, IHC, and IF. For H&E, epidermal thickness was measured at 20 locations per sample, and the mean thickness for each mouse was calculated. IHC and IF staining were performed as previously reported (Luan et al., 2023). Sections were blocked with 3% goat serum and incubated with primary antibodies for 2 h at room temperature. Sections were then developed with DAB for IHC or incubated with fluorophore-conjugated secondary antibodies for IF. Images were acquired, and optical density or fluorescence intensity was quantified using ImageJ (National Institutes of Health, United States).

### PASI-like score

PASI scoring was performed as previously described (Shen et al., 2025). Briefly, disease severity on dorsal skin was evaluated daily using a 3-parameter scale (scaling, erythema, and thickness). Each parameter was scored from 0 to 4 (0, none; 1, mild; 2, moderate; 3, marked; 4, severe). A cumulative score was calculated by summing the three parameters. To ensure objectivity, two independent investigators scored mice separately, and the final score was the mean of the two assessments.

### Cell transfection

Transfection was performed when the cells reached approximately 70% confluence. For small interfering RNA (siRNA) transfection, cells were transfected with siRNA at a final concentration of 50 nM. Briefly, 10 μL of freshly prepared siRNA solution was diluted in Opti-MEM and mixed with 5 μL of transfection reagent (diluted in Opti-MEM) in serum-free medium. The mixture was added to cells and incubated for 48 h. Negative-control siRNA: sense 5′-UUC​UCC​GAA​CGU​GUC​ACG​U-3′; antisense 5′-ACG​UGA​CAC​GUU​CGG​AGA​A-3′. siRNA-PCSK9 sequence: sense 5′-GCC​UGG​AGU​UUA​UUC​GGA​A-3′; antisense 5′-UUC​CGA​AUA​AAC​UCC​AGG​C-3′. For plasmid transfection, 2 μg of plasmid DNA and 5 μL of transfection reagent were each diluted in 250 μL Opti-MEM, combined, and added to cells in serum-free medium for 48 h.

### Quantitative real-time PCR (qPCR)

Total RNA was extracted from mouse skin tissues or HaCaT cells using RNAiso Plus, and RNA concentration was measured using a NanoDrop spectrophotometer (Thermo Fisher Scientific, United States). Single-stranded cDNA was synthesized from 1 µg total RNA using a reverse transcription kit according to the manufacturer’s instructions, and qPCR was performed using SYBR Green PCR Master Mix on a CFX96 real-time PCR system (Bio-Rad, United States). Primer sequences are listed below.

h-*CXCL1* forward: 5′-TCC​TGC​ATC​CCC​CAT​AGT​TA-3′;
h-*CXCL1* reverse: 5′-CTT​CAG​GAA​CAG​CCA​CCA​GT-3′;
h-*CXCL8* forward: 5′-TTT​TGC​CAA​GGA​GTG​CTA​AAG​A-3′;
h-*CXCL8* reverse: 5′-AAC​CCT​CTG​CAC​CCA​GTT​TTC-3′;
h-*S100A8* forward: 5′-ATG​CCG​TCT​ACA​GGG​ATG​AC-3′;
h-*S100A8* reverse: 5′-ACT​GAG​GAC​ACT​CGG​TCT​CTA-3′;
h-*S100A9* forward: 5′-GGT​CAT​AGA​ACA​CAT​CAT​GGA​GG-3′;
h-*S100A9* reverse: 5′-GGC​CTG​GCT​TAT​GGT​GGT​G-3′;
h-*PCSK9* forward 5′-AGA​CCC​ACC​TCT​CGC​AGT​C-3′;
h-*PCSK9* reverse 5′-GGA​GTC​CTC​CTC​GAT​GTA​GTC-3′;
h-*PGR* forward 5′-TTA​TGG​TGT​CCT​TAC​CTG​TGG​G-3′;
h-*PGR* reverse 5′-GCG​GAT​TTT​ATC​AAC​GAT​GCA​G-3′;
h-*ACTB* forward 5′-CGC​GAG​AGA​AGA​TGA​CCC​AGA​TC-3′;
h-*ACTB* reverse 5′-GCC​AGA​GGC​GTA​CAG​GGA​TA-3′;

### RNA-seq analysis

Total RNA was extracted from mouse skin tissues using TRIzol. RNA concentration and integrity were assessed using a NanoDrop ND-1000 spectrophotometer (Gene Company, United States) and an Agilent 2100 Bioanalyzer. RNA purification, reverse transcription, library preparation, and paired-end sequencing were performed by Shanghai Majorbio Bio-pharm Biotechnology Co., Ltd. (Shanghai, China) according to the manufacturer’s instructions. RNA-seq analysis was performed using three independent biological replicates per group. Sequencing was performed on the Illumina NovaSeq 6000 platform with paired-end 150 bp reads. Raw sequencing reads were subjected to quality control and preprocessing to remove adapter sequences, low-quality reads, and reads with excessive ambiguous bases. Clean reads were then used for downstream transcriptomic analysis. Bioinformatic processing was conducted using the NovoMagic Cloud Platform (Novogene, China) and the Majorbio Cloud Platform (Majorbio, China). Gene expression levels were normalized, and differential expression analysis was performed between experimental groups based on predefined thresholds for fold change and statistical significance. Downstream bioinformatic analyses, including functional annotation and pathway enrichment analyses, were performed using the Majorbio Cloud Platform (https://cloud.majorbio.com/page/tools/). The raw RNA-seq FASTQ files and associated metadata have been deposited in the NCBI Sequence Read Archive (SRA) under BioProject accession number PRJNA1509683.

### Western blotting

HaCaT cells were collected and lysed in RIPA buffer supplemented with protease and phosphatase inhibitors. Protein concentration was determined using a BCA assay kit. Proteins were separated by SDS-PAGE and transferred onto polyvinylidene fluoride (PVDF) membranes. The membranes were incubated overnight at 4 °C with primary antibodies, followed by horseradish peroxidase-conjugated secondary antibodies. Signals were detected using a LumiGLO chemiluminescent substrate (Vazyme, China) and visualized with a chemiluminescence imaging system (Bio-Rad, United States).

### Ethics statement

All animal experiments were performed in accordance with the NIH Guide for the Care and Use of Laboratory Animals (National Academies Press, 2011) and were approved by the Experimental Animal Care and Use Committee of Nanjing University (Approval No. IACUC-2601005).

The human skin biopsy specimens used in this study were obtained as de-identified discarded tissue collected during routine diagnostic procedures. Since the specimens were fully anonymized and no identifiable personal information was accessible, the use of these samples for retrospective research purposes was exempt from ethics committee approval according to institutional guidelines.

### Statistical analysis

All statistical analyses were performed using GraphPad Prism (version 8.0; GraphPad Software, La Jolla, CA, United States). Data are presented as mean ± SEM. For comparisons between two independent groups, an unpaired two-tailed Student’s t-test was used. For comparisons among more than two groups, one-way ANOVA followed by Tukey’s multiple comparisons test was used for parametric data, whereas the Kruskal–Wallis test followed by Dunn’s multiple comparisons test was used for non-parametric data. Longitudinal data, including body weight changes and PASI-like scores, were analyzed using repeated-measures two-way ANOVA with time and treatment group as factors, followed by Tukey’s multiple comparisons test where appropriate. When the assumption of sphericity was not met, Geisser-Greenhouse correction was applied. Statistical tests, sample sizes, and P-value annotations are indicated in the corresponding figure legends. Exact P values are reported where appropriate, with values less than 0.001 indicated as P < 0.001. P < 0.05 was considered statistically significant.

## Results

### PCSK9 dysregulation in human psoriasis and the effects of PCSK9 inhibition in an IMQ-induced murine model

To investigate the relationship between PCSK9 and psoriasis, we assessed PCSK9 expression in skin samples from patients with psoriasis. Immunohistochemistry showed that PCSK9 was highly expressed in psoriatic lesions (n = 7) compared with healthy control skin (n = 7) (Figure 1A). Next, using the GSE4602 dataset from the GEO database, we analyzed PCSK9 expression across 64 healthy skin samples, 58 psoriatic lesional samples, and 58 non-lesional samples adjacent to lesions. PCSK9 expression was significantly higher in lesional tissue than in healthy and non-lesional tissue, whereas no significant difference was observed between healthy and non-lesional tissue (Figure 1B). Given prior evidence that PCSK9 deficiency alleviates psoriasis-like inflammation, we used an IMQ-induced model to assess the therapeutic effects of the PCSK9 inhibitor SBC-110736. Female mice were chosen because PCSK9 expression and its metabolic and immunological functions show sex-dependent differences, with females exhibiting greater sensitivity to PCSK9 modulation (Ferri et al., 2020). This allowed evaluation of drug efficacy under a physiologically more responsive condition. After four consecutive days of IMQ treatment, mice developed erythema and scaling on the dorsal skin. H&E staining revealed hyperkeratosis and a markedly thickened stratum corneum in the model group. In contrast, SBC-110736 treatment markedly reduced scaling and erythema and decreased the PASI-like score (Figures 1C,D). During the IMQ-induced psoriasis-like dermatitis model, IMQ-treated mice exhibited a significant reduction in body weight compared with control mice. SBC-110736-treated mice showed a distinct pattern of body weight change during the observation period (Figure 1E). Repeated-measures two-way ANOVA revealed significant treatment × time interactions for both body weight changes and PASI scores, indicating that the longitudinal changes differed among treatment groups (P < 0.001 for body weight and P < 0.001 for PASI scores). However, Tukey’s *post hoc* comparisons showed no significant differences in body weight between the IMQ and IMQ + SBC-110736 groups at individual time points. Immunohistochemistry showed that PCSK9 expression was increased in the skin of IMQ-treated mice and decreased to near-control levels after SBC-110736 treatment (Figure 1F). Immunofluorescence analysis demonstrated that SBC-110736 treatment significantly reduced infiltration by CD4^+^ and CD11b^+^ cells in the skin (Figures 1G,H). Collectively, these results indicate that PCSK9 is upregulated in a murine psoriasis-like model and that pharmacological inhibition of PCSK9 ameliorates IMQ-induced skin inflammation.

**FIGURE 1:**
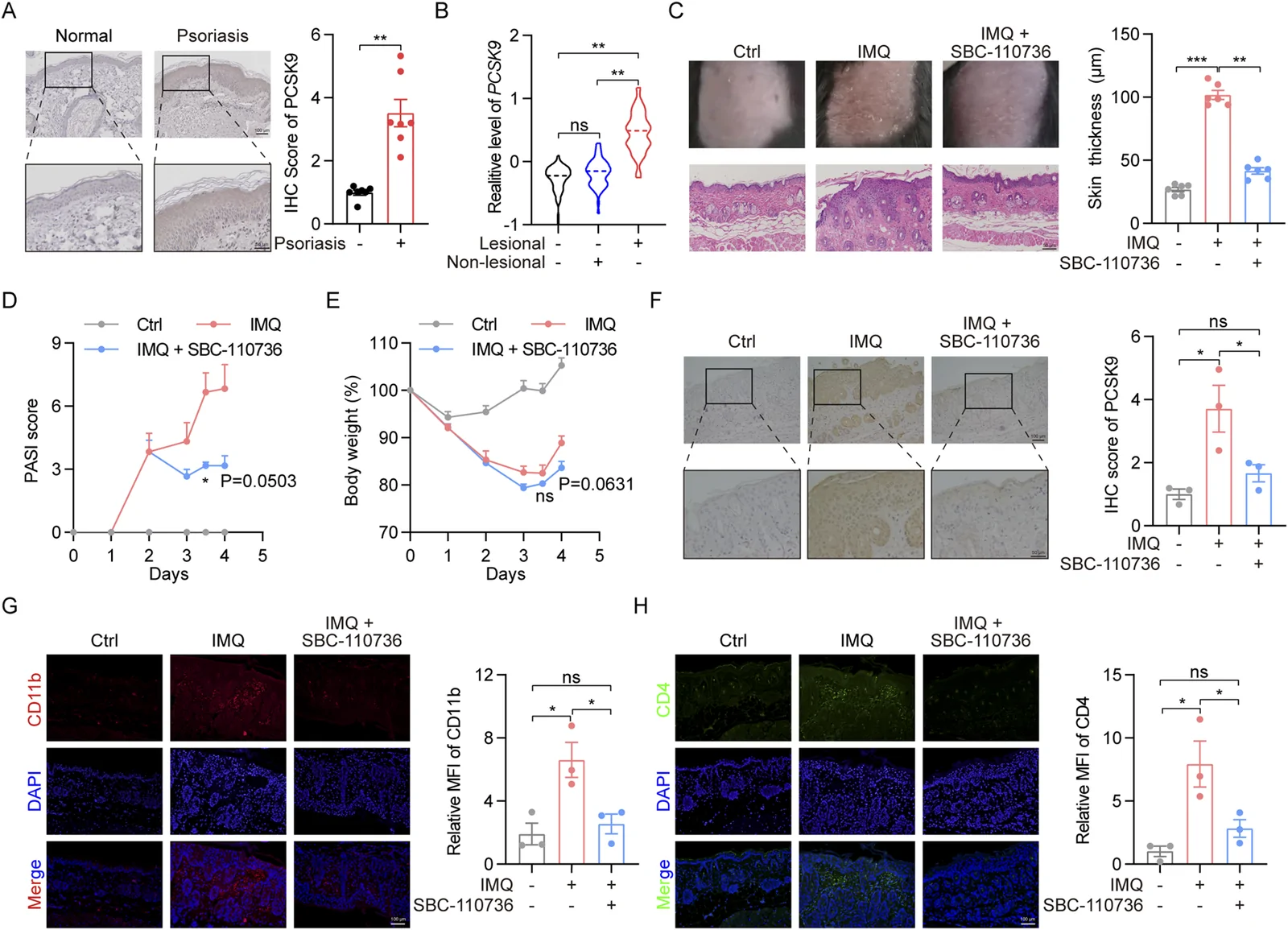
PCSK9 dysregulation in human psoriasis and the effects of PCSK9 inhibition in an IMQ-induced murine model. **(A,B)** PCSK9 expression was evaluated in skin samples from patients with psoriasis and healthy donors. **(A)** Immunohistochemical (IHC) staining for PCSK9 in skin biopsies from patients with psoriasis (n = 7) and healthy donors (n = 7). **(B)** PCSK9 expression levels in lesional and non-lesional skin of patients with psoriasis compared with healthy donor skin. Data are from the microarray dataset GSE4602. **(C–H)** The effects of PCSK9 inhibition were investigated in an IMQ-induced psoriasis-like mouse model. Female C57BL/6J mice (6–8 weeks old) were shaved on the dorsal skin 2–3 days before IMQ application to induce psoriasis-like dermatitis. Mice (n = 6 per group) were then treated with SBC-110736 (10 mg/kg) or vehicle for 4 days. **(C)** Representative macroscopic photographs and corresponding H&E-stained sections (bottom) of dorsal skin from the different treatment groups. **(D)** Quantification of psoriasis-like dermatitis severity (PASI-like score), including scores for erythema, scaling, and skin thickness. Significance symbols shown below the curves indicate comparisons between the IMQ + SBC-110736 group and the IMQ group. **(E)** Changes in body weight during the experiment. Significance symbols shown below the curves indicate comparisons between the IMQ + SBC-110736 group and the IMQ group. **(F)** Immunohistochemical staining for PCSK9 in skin sections from each group. **(G,H)** Immunofluorescence staining for CD11b and CD4 in skin sections from each group. Data are presented as mean ± SEM; symbols represent individual animals or independent biological replicates. Statistical analyses were performed separately for each panel. For **(A)**, statistical significance was determined using an unpaired two-tailed Student’s t-test. For **(B,C,F,G,H)**, statistical significance was determined using one-way ANOVA followed by Tukey’s multiple comparisons test. For **(D,E)**, longitudinal data were analyzed using repeated-measures two-way ANOVA with time and treatment as factors, followed by Tukey’s multiple comparisons test. Geisser–Greenhouse correction was applied when required. *P < 0.05, **P < 0.01, ***P < 0.001; ns, not significant.

### RNA sequencing analysis revealed potential mechanisms by which PCSK9 inhibition exerts therapeutic effects in psoriatic mice

To further investigate the role of PCSK9 in psoriasis, we performed transcriptomic sequencing on skin samples from the animal experiments described above. We identified 4,158 differentially expressed genes (DEGs) in the IMQ group compared with the control (Ctrl) group (1,940 upregulated and 2,218 downregulated). We also identified 1,467 DEGs in the SBC-110736-treated group compared with the IMQ group (728 upregulated and 739 downregulated) (Figure 2A). We first focused on genes that were upregulated in the IMQ group and downregulated after SBC-110736 treatment. A total of 568 genes showed this overlapping pattern (Figure 2B). A heatmap of these shared genes highlighted key inflammatory mediators, including Ccl20, S100a8, Cxcl1, and Il17a (Figure 2C). Kyoto Encyclopedia of Genes and Genomes (KEGG) enrichment analysis indicated that these genes were predominantly enriched in the interleukin-17 (IL-17) signaling pathway (Figure 2D). Gene Ontology (GO) enrichment analysis further showed enrichment for leukocyte cell-cell adhesion, acute inflammatory responses, ERBB signaling, and epithelial cell proliferation (Figure 2E). We also analyzed genes that were downregulated in the IMQ group and upregulated after SBC-110736 treatment and identified 447 overlapping genes, including Ccl27b, Ngfr, and Pgr (Figure 2F). KEGG enrichment analysis showed that these genes were enriched in biosynthesis of unsaturated fatty acids, cytokine-cytokine receptor interaction, and estrogen signaling (Figure 2G). GO enrichment analysis indicated associations with chemical homeostasis, regulation of hormone levels, and responses to lipid stimuli (Figure 2H). Collectively, these transcriptomic analyses suggest that PCSK9 inhibition mitigates IMQ-induced psoriasis-like inflammation by attenuating IL-17-driven signaling while concomitantly restoring hormone- and lipid-metabolism-related pathways, supporting a mechanistic link between PCSK9, hormonal regulation, and psoriatic inflammation.

**FIGURE 2:**
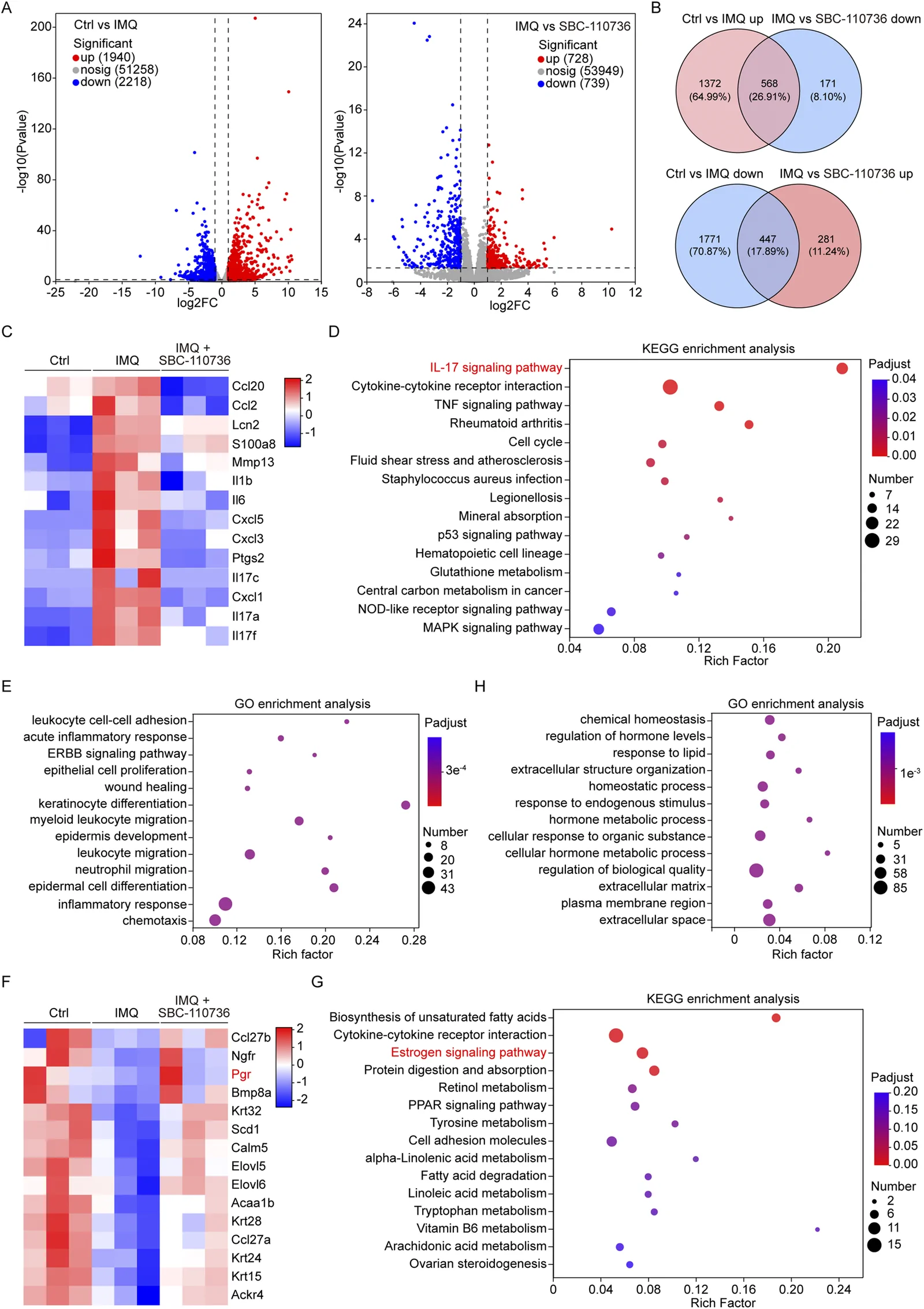
RNA sequencing reveals the potential mechanisms of PCSK9 inhibition in psoriatic mice. **(A)** Volcano plots showing statistically significant differentially expressed genes (DEGs) in the Sham vs. IMQ (left) and IMQ vs. IMQ + SBC-110736 (SBC) (right) comparisons. DEGs were identified using an adjusted P value <0.05 and |log2FC| > 1. **(B)** Venn diagrams of gene overlaps: upregulated in IMQ/downregulated in SBC (top); downregulated in IMQ/upregulated in SBC (bottom). **(C)** Heatmap of shared upregulated genes (top, Figure 2B) across samples. The color scale from red to blue indicates high to low expression, respectively. **(D)** KEGG enrichment analysis. **(E)** GO enrichment analysis. **(F)** Heatmap of shared downregulated genes (bottom, Figure 2B). **(G,H)** KEGG and GO enrichment analyses, respectively.

### PCSK9 decreases PR expression possibly via regulation of its upstream transcription factor

Given the prominent upregulation of Pgr among genes restored by SBC-110736 treatment, we next investigated whether progesterone receptor (PR) signaling mediates the protective effects of PCSK9 inhibition in psoriasis. To this end, we examined PR expression in psoriatic skin. Immunohistochemistry showed that PR expression was markedly reduced in psoriatic lesions compared with healthy control skin (Figure 3A). Consistently, analysis of the GSE4602 dataset showed that PR expression was significantly lower in lesional tissue than in healthy and non-lesional skin (Figure 3B). Immunohistochemistry showed that PR expression was decreased in the skin of IMQ-treated mice and increased to near-control levels after SBC-110736 treatment (Figure 3C). Next, we overexpressed PCSK9 in HaCaT cells and quantified PR expression at the mRNA and protein levels. The overexpression of PCSK9 significantly reduced PR mRNA and protein abundance (Figures 3D,E). Conversely, PCSK9 knockdown markedly increased PR expression (Figures 3F,G). Together, these data support an inverse relationship between PCSK9 and PR expression in keratinocytes. We next examined transcription factors associated with the PGR gene. Reported PR transcriptional regulators include HOXA5, C/EBPβ, C-JUN, and RNPC1 (Raman et al., 2000; Wang et al., 2018; Petz et al., 2004; Lou et al., 2017). To predict candidate transcription factors regulating human and murine PGR, we performed *in silico* screening using the JASPAR, PROMO, GTRD, HumanTFDB, and AnimalTFDB databases. Because the candidate transcription factors predicted by JASPAR and PROMO differed substantially, we analyzed the two prediction sets separately. Specifically, we intersected PROMO or JASPAR predictions with results from GTRD and HumanTFDB/AnimalTFDB to obtain overlapping candidates (Figures 3H,I). Using our transcriptomic data, we generated heatmaps to visualize expression patterns of the overlapping transcription factor candidates (Figures 3J,K).

**FIGURE 3:**
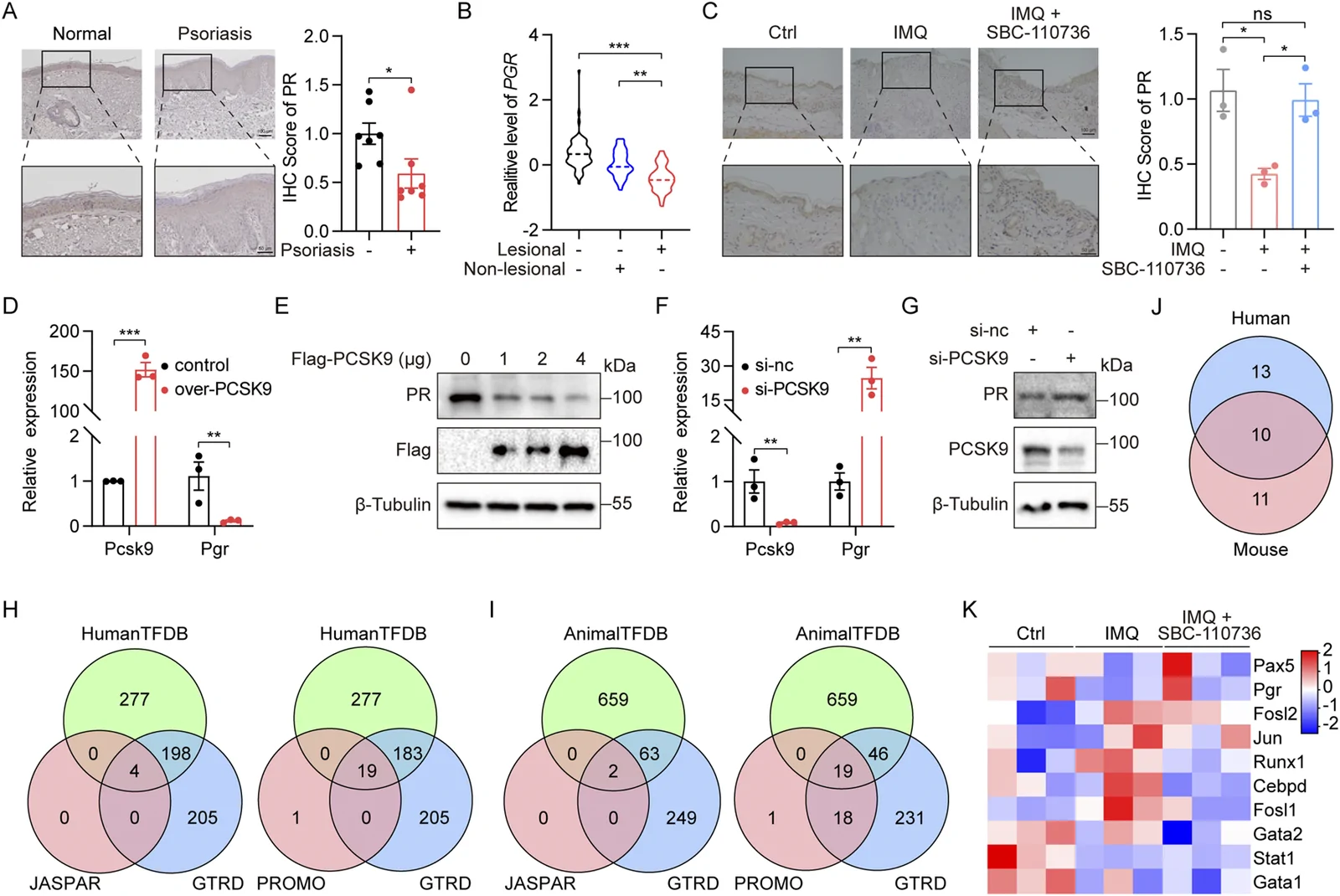
PCSK9 decreases PR expression possibly via regulation of its upstream. **(A)** Immunohistochemical (IHC) staining for PR in skin biopsies from patients with psoriasis (n = 7) and healthy donors (n = 7). **(B)** PR expression levels in lesional and non-lesional skin of patients with psoriasis compared with healthy donor skin. Data are from the microarray dataset GSE4602. **(C)** Immunohistochemical staining for PR in skin sections from each group. **(D,E)** Changes in PR gene and protein levels after PCSK9 overexpression. **(F,G)** Changes in PR gene and protein levels after PCSK9 knockdown (si-PCSK9). **(H)** Predicted transcription factors from the human database. **(I)** Predicted transcription factors from the mouse database. **(J,K)** Intersection of human and mouse predictions and a heatmap of animal transcriptome samples. Data are presented as mean ± SEM; symbols represent individual animals or independent biological replicates. Statistical analyses were performed separately for each panel. For **(A,D,F)**, statistical significance was determined using an unpaired two-tailed Student’s t-test. For **(B,C)**, statistical significance was determined using one-way ANOVA followed by Tukey’s multiple comparisons test. *P < 0.05, **P < 0.01, ***P < 0.001; ns, not significant.

### PR agonist attenuated a psoriasis-like phenotype in the IMQ-induced murine model via inhibiting IL-17 signaling pathways

Next, we investigated whether activation of PR signaling could alleviate IMQ-induced psoriasis-like skin inflammation in mice. Progesterone (Pg), a PR agonist, was applied topically in the animal model (Meng et al., 2019; Waddell and O'Leary, 2002). Male mice were used to avoid estrous-cycle fluctuations of endogenous hormones in females, ensuring a stable endocrine background for assessing progesterone-specific effects. Although repeated-measures two-way ANOVA showed significant overall effects of time, treatment, and treatment × time interaction on body weight changes, Tukey’s *post hoc* comparisons showed no significant difference between the IMQ and IMQ +0.3% Pg groups at individual time points (Figure 4A). Topical application of 0.1% Pg significantly reduced scaling, erythema, and PASI-like scores in IMQ-treated mice, whereas increasing the concentration to 0.3% did not further enhance the therapeutic effect. Repeated-measures two-way ANOVA followed by Tukey’s multiple comparisons test showed that 0.1% Pg significantly reduced PASI-like scores compared with the IMQ group at later time points, while no significant difference was observed between the 0.1% and 0.3% Pg groups (Figures 4B,C). These results indicate that Pg effectively attenuates IMQ-induced psoriasis-like skin lesions. Immunohistochemical analysis further confirmed that PR agonist treatment significantly reduced the expression of PCSK9, phospho-NF-κB p65 (p-p65), Ki-67, and PCNA in skin tissues, suggesting that PR activation suppresses downstream inflammatory signaling and keratinocyte proliferation (Figure 4D). Consistently, immunofluorescence staining showed that Pg treatment markedly diminished the infiltration by CD4^+^ and CD11b^+^ immune cells in the skin of IMQ-treated mice (Figure 4E).

**FIGURE 4:**
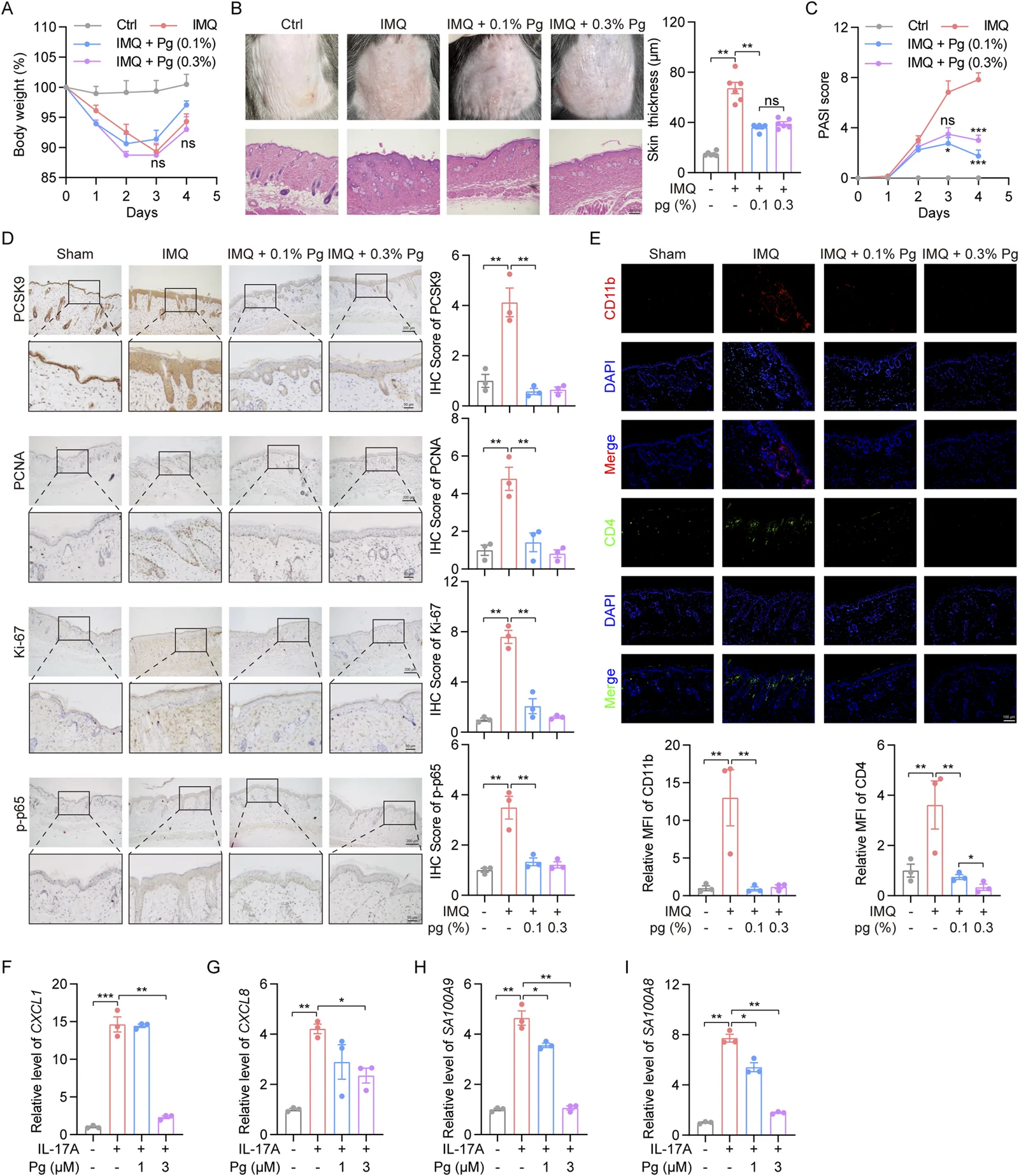
PR agonist alleviates psoriasis-like skin inflammation and suppresses IL-17 pathways. Male C57BL/6J mice (6–8 weeks old) were shaved on the dorsal skin 2–3 days before IMQ application to induce psoriasis-like dermatitis. Mice (n = 6 per group) were then treated with progesterone or vehicle for 4 days. **(A)** Changes in body weight during the experiment. Significance symbols shown below the curves indicate comparisons between the IMQ + Pg (0.3%) group and the IMQ group. **(B)** Representative macroscopic photographs and corresponding H&E-stained sections (bottom) of dorsal skin from the different treatment groups. **(C)** Quantification of psoriasis-like dermatitis severity (PASI-like score), including scores for erythema, scaling, and skin thickness. Significance symbols shown above the curves indicate comparisons between the IMQ + Pg (0.3%) group and the IMQ group, whereas symbols shown below the curves indicate comparisons between the IMQ + Pg (0.1%) group and the IMQ group. **(D)** Immunohistochemical staining for PCSK9, PCNA, Ki-67, and p-p65 in skin sections from each group (n = 3). **(E)** Immunofluorescence staining for CD11b and CD4 in skin sections from each group (n = 3). **(F–I)** Quantitative PCR analysis of mRNA levels (*CXCL1*, *CXCL8*, *S100A8*, and *S100A9*) in HaCaT cells treated with IL-17A and progesterone. Results were normalized to *ACTB* (n = 3). Data are presented as mean ± SEM; symbols represent individual animals or independent biological replicates. Statistical analyses were performed separately for each panel. For **(B,D,E,F,G,H,I)**, statistical significance was determined using one-way ANOVA followed by Tukey’s multiple comparisons test. For **(A–C)**, longitudinal data were analyzed using repeated-measures two-way ANOVA with time and treatment as factors, followed by Tukey’s multiple comparisons test. Geisser–Greenhouse correction was applied when required. *P < 0.05, **P < 0.01, ***P < 0.001; ns, not significant.

Next, we performed *in vitro* validation using HaCaT keratinocytes to determine whether PR activation functionally modulates IL-17A–driven signaling. Consistent with our transcriptomic findings, treatment with Pg significantly blunted IL-17A-induced inflammatory activation in HaCaT cells. At the transcriptional level, PR activation significantly suppressed the induction of multiple IL-17-responsive signature genes. In particular, the expression of *CXCL1* and *CXCL8*, two potent neutrophil-attracting chemokines, was significantly reduced (Figures 4F,G). Likewise, the mRNA levels of *S100A8* and *S100A9*, hallmark antimicrobial peptides that are strongly upregulated in psoriatic inflammation, were significantly decreased upon PR agonist treatment (Figures 4H,I). Collectively, these findings show that PR activation dampens IL-17A-triggered inflammatory transcriptional programs in keratinocytes, reinforcing the notion that PR serves as an endogenous regulatory checkpoint that restrains IL-17–mediated cutaneous inflammation in keratinocytes.

## Discussion

This study suggests that the PCSK9-PR-IL-17 axis may represent a previously underappreciated regulatory pathway involved in psoriatic inflammation. While PCSK9 is widely recognized as a systemic regulator of lipid metabolism, our data indicate that increased PCSK9 expression in psoriatic skin is associated with reduced progesterone receptor expression and dysregulated cutaneous immune homeostasis. The skin is not merely a passive barrier but a functionally active endocrine organ, where PR signaling may contribute to immune regulation and inflammatory restraint. Clinical observations, such as improvement of psoriasis in some patients during pregnancy and disease worsening during menopause or progesterone-deficient conditions, are consistent with the possibility that PR-related signaling may influence inflammatory activity in psoriasis (Murase et al., 2005). In our experimental models, PCSK9 inhibition or PR activation was associated with reduced IL-17-related inflammatory responses, suggesting a functional link among PCSK9, PR signaling, and IL-17-driven inflammation.

Importantly, our findings provide potential therapeutic implications beyond the mechanistic role of PCSK9 in psoriasis. The ability of the small-molecule PCSK9 inhibitor SBC-110736 to ameliorate psoriasis-like phenotypes suggests that PCSK9 inhibition may represent a potential therapeutic strategy for modulating cutaneous inflammation. Given the well-established association between psoriasis and systemic comorbidities, including metabolic and cardiovascular disorders, our findings raise the possibility that PCSK9-targeted therapies may have broader therapeutic implications. However, whether PCSK9 inhibition can influence these systemic manifestations remains to be determined. Further studies, including comprehensive metabolic evaluations, cardiovascular assessments, and clinical investigations, are warranted to clarify the translational potential of PCSK9-targeted therapies in psoriasis. This perspective is particularly relevant in the context of the “psoriatic march,” a proposed model in which chronic cutaneous inflammation contributes to the development of systemic inflammatory and vascular complications (Boehncke et al., 2011). Moreover, the effectiveness of topical progesterone further supports the potential for localized modulation of the PCSK9–PR–IL-17 axis, which may reduce systemic exposure and related side effects.

Nevertheless, several limitations should be acknowledged. Although our findings support a functional association among PCSK9, PR, and IL-17 signaling in psoriatic inflammation, whether this axis represents a direct linear causal pathway requires further investigation. While our study demonstrates an inverse relationship between PCSK9 and PR in keratinocytes, the precise transcriptional or post-translational mechanisms through which PCSK9 regulates PR expression remain unclear. Future studies involving protein stability assays, ubiquitination analysis, proteasome inhibition experiments, and chromatin immunoprecipitation (ChIP) will be needed to further investigate the underlying mechanism. Additionally, the contribution of immune cells, particularly IL-17-producing T cells and macrophages, to this axis should be examined in keratinocyte-specific knockout models. Future studies should also assess whether PCSK9 levels correlate with disease severity or therapeutic response in psoriasis patients, which would strengthen the clinical translatability of our findings. Whether progesterone-mediated anti-inflammatory effects are strictly PR-dependent also remains to be determined. Future studies using PGR knockdown or PR antagonism with appropriate treatment controls will be needed to clarify this issue.

From a translational perspective, the therapeutic potential of targeting the PCSK9-PR-IL-17 axis requires cautious interpretation. Neither SBC-110736 nor progesterone is currently approved for psoriasis treatment. SBC-110736 is an experimental small-molecule PCSK9 inhibitor, and its approved indications, therapeutic dose range, pharmacokinetic properties, and long-term safety profile have not been established. Therefore, SBC-110736 was used in this study as a pharmacological tool to explore the role of PCSK9 in psoriasis-like inflammation rather than as a clinically validated therapeutic agent. Progesterone is clinically used mainly for gynecological and reproductive endocrine indications, but its repurposing for psoriasis would require careful evaluation of hormone-related adverse effects, including endocrine and reproductive effects, fluid retention, mood-related symptoms, and thromboembolic or cardiovascular risks in susceptible individuals. Thus, future studies are required to define the efficacy, dosing strategy, pharmacokinetics, contraindications, and long-term safety of these interventions before clinical translation.

In conclusion, our study suggests that PCSK9 inhibition may restore PR-mediated suppression of IL-17 signaling and thereby attenuate psoriasis-like inflammation. These findings identify the PCSK9-PR-IL-17 axis as a potential regulatory pathway linking metabolic and hormonal signaling with cutaneous inflammation. Further investigation of this pathway may provide new insights into the pathophysiology and therapeutic modulation of inflammatory dermatoses, although therapeutic translation of PCSK9-targeted or progesterone-based interventions for psoriasis requires further preclinical validation and clinical safety evaluation.

## Data Availability

The data presented in the study are deposited in the NCBI Sequence Read Archive (SRA) repository, under BioProject accession number PRJNA1509683, available at https://www.ncbi.nlm.nih.gov/bioproject/PRJNA1509683.
